# Relativistic torques induced by currents in magnetic materials: physics and experiments

**DOI:** 10.1039/c8ra04001j

**Published:** 2018-07-12

**Authors:** Bhaskar Kaviraj, Jaivardhan Sinha

**Affiliations:** Department of Physics, School of Natural Sciences, Shiv Nadar University Gautam Budh Nagar 203207 Uttar Pradesh India bhaskar.kaviraj@snu.edu.in; Department of Physics and Nanotechnology, SRM Institute of Science and Technology Kattankulathur 603203 Tamil Nadu India

## Abstract

In this review article, an insight of the physics that explains the phenomenon of torques induced by currents in systems comprising ferromagnetic (FM)–non-magnetic (NM) materials has been provided with particular emphasis on experiments that concern the observation of such torques. An important requirement of systems that enables observation of such relativistic torques is that the material needs to possess large spin–orbit coupling (SOC). In addition, the FM/NM interface should be of high quality so that spin angular momentum can be transferred across the interface. Under such conditions, the magnetization of a magnetic material experiences a torque, and can be reversed, thanks to the phenomenon of the spin Hall effect in the NM layer with large SOC. A reciprocal process also occurs, in which a changing magnetization orientation can produce spin current, *i.e.* current that supports spin angular momentum. It is important to know how these processes occur which often tells us about the close connection between magnetization and spin transport. This paves the way to transform technologies that process information *via* magnetization direction, namely in magnetic recording industry. This field of physics being relatively young much remains to be understood and explored. Through this review we have attempted to provide a glimpse of existing understanding of current induced torques in ferromagnetic thin film heterostructures along with some future challenges and opportunities of this evolving area of spintronics. Specifically, we have discussed the state-of-the art demonstrations of current-induced torque devices that show great promise for enhancing the functionality of magnetic memory devices.

## Abstract 2

The article focuses on the physics that explains the phenomenon of torques induced by currents in systems comprising ferromagnetic–non-magnetic materials with particular emphasis on experiments that concern the observation of such torques.

## Graphical abstract

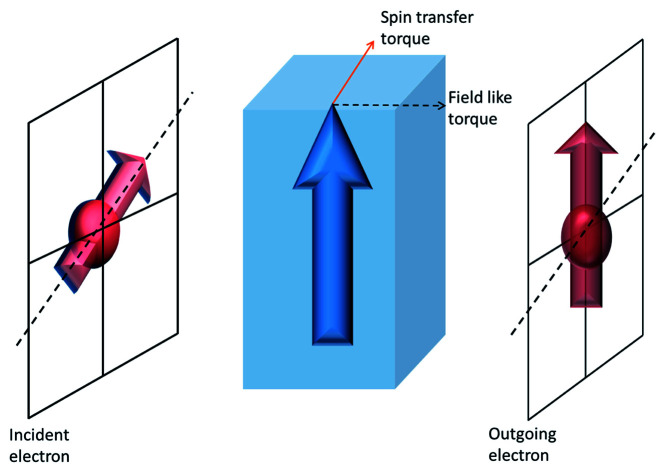

## 1. Introduction

Magnetic polarization or magnetization is an inherent property of ferromagnets that have been long used to store information. In classical electromagnetism, magnetization is a vector field that represents the density of permanent or induced magnetic dipole moments in a magnetic material. The origin of magnetic moments responsible for magnetization can either be due to microscopic electric currents resulting from the motion of electrons in atoms or due to spin of the electrons or the nuclei. Net magnetization results from the response of a material to an external magnetic field, together with any unbalanced magnetic dipole moments that may be inherent in the material itself; for example, in ferromagnets. The direction of magnetization of a ferromagnet may be used to represent a bit of information, *e.g.*, a spin-up orientation may be represented as 1 and a spin-down as 0. Often these are used in magnetic storage devices. Usually, external applied magnetic fields, *e.g.* Oersted field from current through wires have been used to alter the direction of magnetic moments. In the past few years, phenomena such as spin-transfer torque and spin–orbit torques have enhanced the possibility of manipulating magnetization of ferromagnets in the absence of any external applied magnetic field. Such discoveries have revolutionized the scientific and technological development of magnetic storage devices. Another important feature of these “all electronic” magnetic devices is that they can be functionalized and integrated to the existing semiconductor based electronic devices.

In a classical picture, while the electric current is known to be accompanied with moving charges, the spin current however, is understood in terms of transfer of angular momentum. The angular momentum carried by the spin current can be transferred to the magnetization near its vicinity. This phenomenon, known as spin-transfer was first put into theory by Slonczewski and Berger.^1,2^ The interaction between the itinerant electrons in a ferromagnet that are spin-polarized and its magnetization gives rise to this torque. The interaction however is a local one, existing only in regions where spin currents flow, and can be very strong. Under the influence of charge current such torques have been found to be present in most of the magnetic materials, transition metal ferromagnets, magnetic semiconductors, oxide ferromagnets and also in antiferromagnets. They are also found to occur at interfaces of insulating magnetic materials. Various structures and device geometries, including nanopillars and point contacts of multilayers of magnetic-non-magnetic materials as well as nanowires under the influence of spin transfer torque have led to an in-depth understanding of physics issues pertaining to applications of spintronic devices.

This article reviews the fundamentals and phenomena of devices based on current induced relativistic torques in various materials which falls currently into the core of a rapidly advancing field of current induced magnetization dynamics. The article discusses about how these torques will help in miniaturizing devices for use in magnetic random access memories (MRAM), as compared to the use of magnetic fields to reorient magnetization to store information. We also discuss about a new way of probing spin transport – that is, spin pumping-a reciprocal process to that of spin torques involving the emission of spin currents by magnetization reorientation. Remarkable developments in both these phenomena – spin torques and spin pumping have opened up possibilities for the improvement of nanometer-scale electronic devices.

**Fig. 1:**
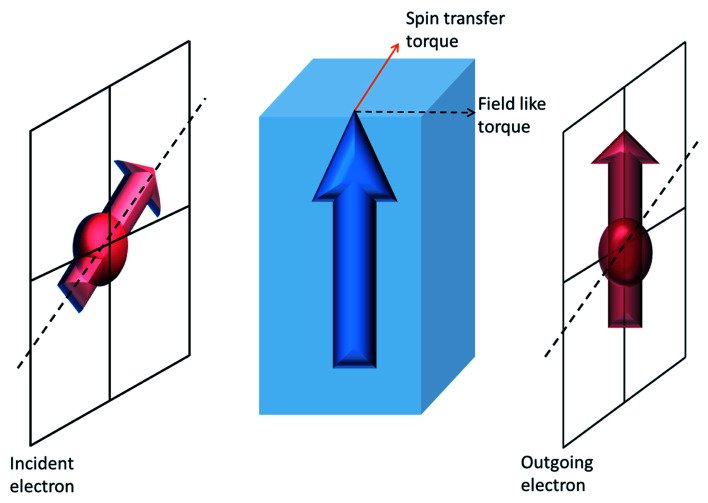
Illustration of current-induced torques. The interaction of spin polarized conduction electron with the magnetization of a ferromagnetic material causes a change in the direction of spin polarization of the outgoing electron. The difference in spin polarization results in a torque on the magnetization of the ferromagnetic layer. The torque acting in the ‘plane of incident and outgoing spin direction’ is called a spin-transfer torque whereas the torque perpendicular to this plane is called a ‘field-like’ torque. The bold blue arrow represents magnetization vector of the ferromagnet.

Whenever there is an imbalance in the flow of up-spin and down-spin electrons through a material, there is net flow of spin angular momentum (spin current) across it. The conduction electron spin now interact with the magnetization of the magnetic material which results in a reorientation of its spin after transmission (or reflection from) through the material. According to conservation of angular momentum, a change in the conduction electron spin orientation (spin angular momentum) must result in a torque on the magnetization of the ferromagnet. This torque can have two directions – with respect to the plane of incident and outgoing electron spin. A torque which acts in that plane is called spin-transfer (or adiabatic) torque and that which is along the direction perpendicular to the plane is called a field-like (or non-adiabatic) torque (*cf.*Fig. 1). The magnitude of these two spin-torques is dependent on the material and device structure. Material properties determine the threshold currents for magnetization switching and magnetization precession frequency.

## 2. Origin of spin–orbit coupling

An electron, having a linear momentum *p*, experiences a Lorentz force in the presence of a magnetic field *B*, the direction of the force being perpendicular to its motion ***F⃑*** = −e***p⃑*** × ***B⃑***/*m*. The electron possess Zeeman energy *μ*_B_***σ⃗***·***B⃑***, where ***σ⃗*** are the Pauli spin matrices vector, *m* and *e* are mass and charge of the electron respectively, and *μ*_B_ = 9.27 × 10^−24^ J T^−1^ is the Bohr magneton. When this electron moves in an electric field ***E***, it experiences, in its own rest-frame, an effective magnetic field ***B***_**eff**_ ∼ ***E*** × ***p***/*mc*^2^. The effective magnetic field ***B***_**eff**_ induces a momentum-dependent Zeeman energy called the spin–orbit (SO) coupling given by *H*_SO_ ∼ *μ*_B_(***E*** × ***p***)·***σ***/*mc*^2^. In crystals, it is the gradient of the crystal potential that determines the electric field, ***E*** = −**∇*V***, which produces a SO field ***w***(***p***) = −*μ*_*B*_(**∇*V*** × ***p***)/*mc*^2^. This odd-in-p spin–orbit field tends to survive in systems that lack spatial inversion symmetry.

### 2.1. Dresselhaus and Rashba spin orbit coupling

Dresselhaus^3^ first noticed that in III–V compounds such as GaAs or InSb possessing zinc-blende type structures lacking a center of inversion, the SO coupling close to the *Γ* point have the form1
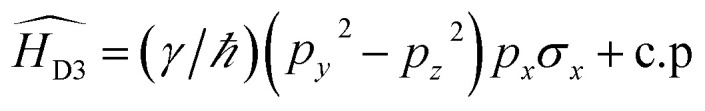
where c.p denotes cyclic permutation of indices.

The cubic Dresselhaus SO coupling given in eqn (1) reduces to the linear Dresselhaus SO coupling in the presence of strain along (001) direction.2
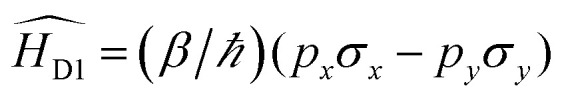
where *β* = *γp*_*z*_^2^

With broken structural inversion symmetry in quantum wells along the growth direction, it was first proposed by Vas'ko,^4^ Bychkov and Rashba^5^ that the interfacial electric field ***E*** = *E*_*z*_***ẑ*** results in a SO coupling of the form3
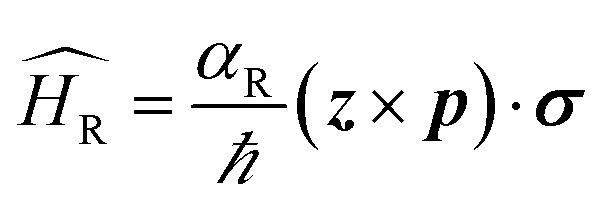
where *α*_R_ is the Rashba parameter. Theoretical investigations indeed reveal that lack of inversion symmetry does not only create an additional electric field *E*_*z*_, but also distorts the electron wave-function close to the nuclei where the plane–wave approximation no longer works.

**Fig. 2:**
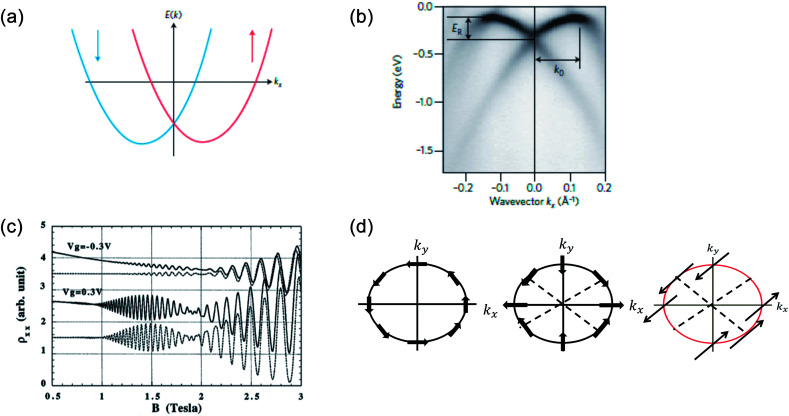
Rashba spin splitting at interfaces. (a) Energy dispersion of a one-dimensional free electron gas where spin-momentum locking is induced by Rashba spin–orbit coupling. The red and blue arrows represent two bands carrying opposite spin momenta. (b) Energy dispersion at the surface of a BiAg (111) alloy measured by ARPES.^12^ The energy shift *E*_R_ and momentum offset *k*_0_ clearly confirms Rashba splitting. (c) Longitudinal resistance as functions of applied magnetic field in the presence of different gate voltages in InAs/InGaAs quantum wells. Solid lines are experimental data and dotted lines are simulations. (d) Spin texture due to Rashba (left) and linear (middle) Dresselhaus SO coupling. The strain is applied along [001]. When both Rashba and Dresselhaus SO coupling are present with equal magnitude, the SO field (shown by arrows) aligns along [110]. Figure reproduced with permission from: (a) ref. 100, *Nature Materials* (b) ref. 12, APS; (c) ref. 7, APS.

Both Dresselhaus and Rashba spin–orbit coupling lock spin to the linear momentum and the consequence is that the spin-bands in energy are split (Fig. 2(a)). In certain metallic surfaces also, such band splitting has been observed (Fig. 2(b)). Fig. 2(d) illustrates the spin texture at the Fermi surface in the case of Rashba (left) and Dresselhaus (*p*-linear) (middle) spin–orbit coupling. When magnitudes of both types of SO coupling are equal, the spin–orbit field aligns along [110] direction which results in the suppression of spin relaxation along this direction (right).^6^

### 2.2. Measurements of Rashba spin orbit coupling

In a wide range of materials that present either interfacial or bulk inversion asymmetry, the Rashba parameter has been calculated. In InAlAs/InGaAs two-dimensional semiconductor heterostructures^7,8^ (Fig. 2(c)), the Rashba parameter calculated (∼0.67 × 10^−11^ eV m) was found to be comparable to that of SrTiO_3_ (001) single crystals with the help of weak localization measurements (∼0.5 × 10^−11^ eV m).^9^ At the surface of heavy metals such as Au,^10^ Ir^11^ or 
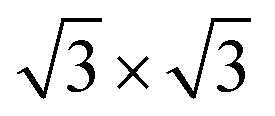
 BiAg (111) alloys,^12^ the Rashba spin orbit coupling parameter was found to be two orders of magnitude larger than in semiconductors (∼3.7 × 10^−10^ eV m for BiAg (111) alloy). Recently, it has been found that topological insulators also exhibit comparable Rashba parameters^13^ (∼4 × 10^−10^ eV m for Bi_2_Se_3_). Evidences of Rashba-type splitting of band structure in systems where bulk inversion asymmetry is broken has been found in materials such as BiTeI, a polar semiconductor where SOC parameter was found to be ∼3.85 × 10^−10^ eV m. This is as large as that found on topological insulator surfaces. Finally, in interfaces of Fe/GaAs^14^ and IrMn/MgO,^15^ large SO coupling has been reported from tunneling anisotropic magnetoresistance measurements, which is usually a change in tunneling resistance as a function of magnetization direction.

## 3. Spin galvanic effects and spin torques

That the spin Hall effect (SHE) is not the only possible mechanism for torques induced by lateral currents in ferromagnet–normal metal (FM–NM) bilayers, have been realized from early experiments with relativistic torques.^16^ If in such systems, the interface breaks the structural inversion symmetry, it means that spin Hall effect induced spin torque must be accompanied by some other microscopic mechanism. The origin can be traced back to spin galvanic phenomena that were explored earlier in inversion-asymmetric normal metals.^17^ In the picture of SHE, a spin current generated in the normal metal is absorbed in the ferromagnetic layer and induces a STT. In a competing scenario, inversion asymmetric surfaces create non-uniform density of spins in the presence of an electric current, a process known as inverse spin galvanic effect (ISGE).^18–23^ A spin–orbit torque (SOT) is directly induced due to the coupling of the magnetic moments in FM and the carrier spins.^24–26^

Spin Hall effects and inverse spin galvanic effects, both allowing for electrical alignment of spins in the same structure, are known to complement each other.^19,21,22,27^ It is important to understand the subtle roles that these two processes play, not only from basic physics, but also from application point of view. In analogy to a galvanic (voltaic) cell, where a chemical reaction produces electric current, it is the spin polarization that generates an electrical current (voltage) in spin galvanic effect (SGE). Conversely, an electrical current (voltage) generates spin polarization in the ISGE.

**Fig. 3:**
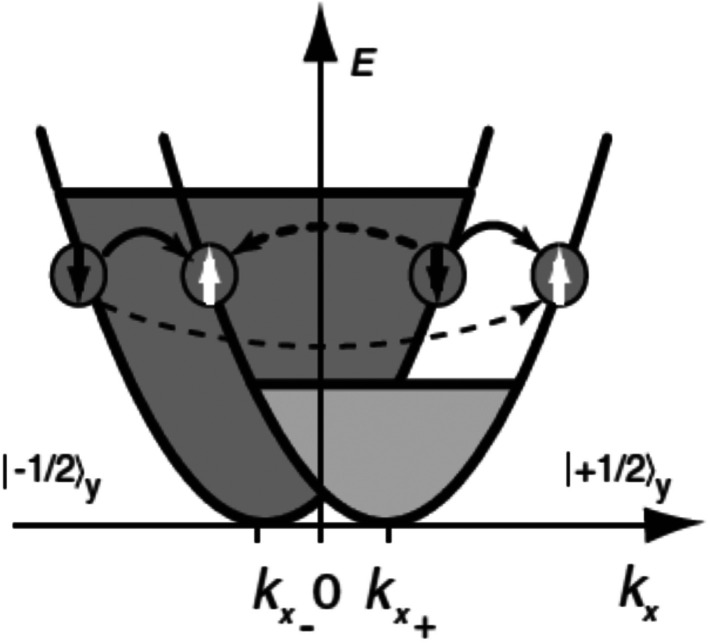
The presence of ***k***-linear terms in electron Hamiltonian explaining the microscopic origin of spin galvanic current. The *σ*_*y*_*k*_*x*_ term in the Hamiltonian splits the conduction band into two parabolas with the spin ±1/2 in the *y* direction. In the event of one spin subband being occupied preferentially, there is a net current along *x* direction due to asymmetric spin-flip scattering (indicated by broken arrows). The final and initial ***k*** vectors determine the rate of spin flip scattering. Four distinct spin-flip scattering are possible as indicated in the figure. Transitions sketched by dashed arrows yield an asymmetric occupation of both sub-bands and hence a current flow. Figure reproduced with permission from Ganichev *et al.*, (2002).^34^

After a lot of theoretical predictions,^28–33^ SGE was initially observed in 2DEG GaAs quantum well.^34^ SGE manifests itself by the production of an electric current in the presence of a non-equilibrium uniform polarization of electron spins. The microscopic origin of SGE effect (described in Fig. 3 caption) is as follows. It is very well-known that spin degeneracy of spin-up and spin-down bands in semiconductor quantum well structures can be lifted due to presence of ***k***-linear terms in the Hamiltonian resulting from spin–orbit interaction in asymmetric potentials. For a two-dimensional electron gas (2DEG) systems, it leads to the electron energy band being splitted into two sub-bands which are shifted in ***k***-space. Each sub-band represent states with spin-up or spin-down. In terms of band structure, spin polarization implies that one sub-band is occupied up to higher energies than the other. No current would flow as long as the carrier distribution in each sub-band is symmetric around the minimum at *k*_*x*∓_. In the event of asymmetric spin relaxation events (dashed arrows in Fig. 3), a net current flow results which means that a current is driven by homogenous spin polarization.

**Fig. 4:**
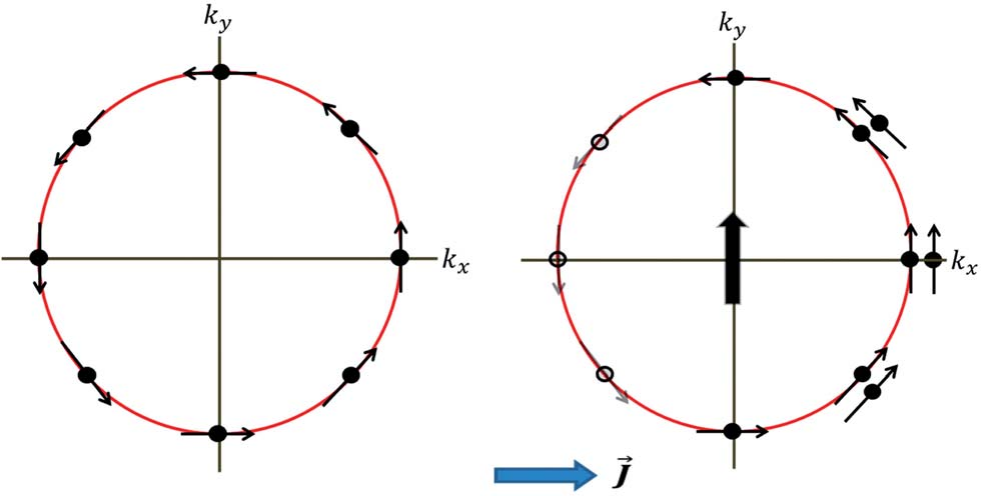
Left panel: Rashba spin texture in equilibrium with zero net spin density. Right panel: An applied electric current (*J*) results in non-equilibrium redistribution of spin eigen states, and therefore a non-zero spin density (vertical arrow) is developed due to broken inversion symmetry of spin texture.

The microscopic picture of ISGE is illustrated in Fig. 4. The presence of electric current causes a uniform non-equilibrium spin density (vertical arrow in Fig. 4, right panel) due to redistribution of carriers on the Fermi surface in such a way that the spin texture has a broken inversion symmetry. In the presence of Rashba spin–orbit coupling (as illustrated in Fig. 4), this gives rise to a uniform in-plane spin polarization which is perpendicular to the applied current. As a comparison, Fig. 4 (left panel) illustrates Rashba spin texture in equilibrium state with no broken inversion symmetry.

Initially, observations of ISGE, in parallel with SHE were obtained with Kerr–Faraday magneto-optical detection methods or circularly polarized luminescence.^17–23,27^ Wunderlich *et al.*^21,22^ (2004, 2005) was able to detect both the effects in a single two-dimensional hole gas in a AlGaAs/GaAs heterostructure whereas Kato *et al.* observed SHE and ISGE in a n-doped bulk InGaAs sample.^19,27^ Subsequently, Bernevig and Vafek in 2005 predicted,^24^ before the experimental verification by Chernyshov *et al.*,^25^ that ISGE can generate relativistic spin–orbit torque in a ferromagnetic semiconductor thin film of (Ga, Mn)As with broken inversion symmetry caused by straining. Both SHE and ISGE have been found to contribute to relativistic spin torques.^16,26,35–40^

It is worth noting here that spin Hall effects and Mott scattering of free electron beams can have the same extrinsic skew-scattering origin. In condensed matter systems, SHE can arise from the spin-dependent transverse deflection induced by the intrinsic SOC in a perfect crystal with no impurities. The origin of SGE however, commonly observed in non-magnetic materials, is extrinsic. Nevertheless, the basics of spin Hall effect, anomalous Hall effect, spin galvanic and relativistic spin torques can be nicely intertwined, even when considering intrinsic effects.

**Fig. 5:**
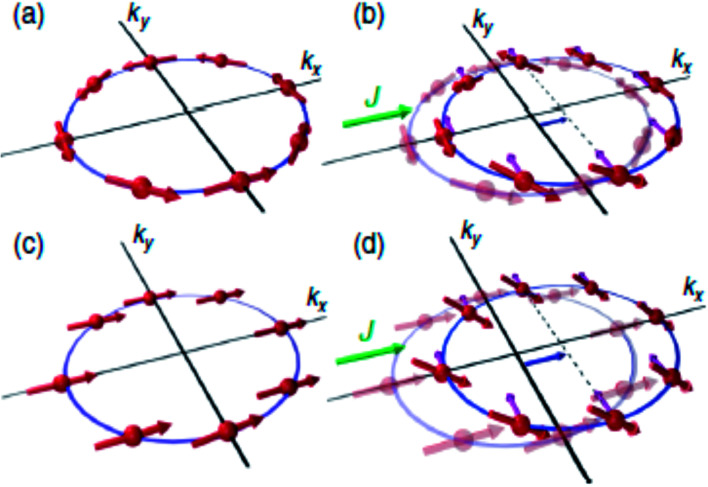
(a) Model of a spin texture in a two-dimensional Rashba spin–orbit coupled system. The spins represented by thick arrows point perpendicular to the momentum. The electric current (*J*) applied along *x*-direction displaces the Fermi surface along the same direction. Electrons moving in momentum space experience additional spin–orbit field represented by thin arrows. Due to current induced field, electron spins tilt up for *k*_*y*_ > 0 and down for *k*_*y*_ < 0, thus creating a spin current along *y*-direction. (c) Spin texture of a 2D Rashba spin–orbit coupled system in presence of an exchange field larger than spin–orbit field. Here all spins align along the direction of exchange field. (d) The same mechanism as in (b) generates uniform, out-of-plane spin polarization. Figure reproduced with permission from Sinova *et al.*, (2004)^50^ and Kurebayashi *et al.*, (2014).^41^

Fig. 5 illustrates that the same current-induced mechanism that generates transverse spin current in the phenomenon of SHE can also induce a spin polarization that is uniform for two specific cases of broken spatial inversion symmetry and broken time reversal symmetry, respectively. Kurebayashi *et al.*,^41^ identified the relativistic spin–orbit torque caused by the non-equilibrium uniform spin polarization of this intrinsic origin in the ferromagnetic semiconductor of (Ga, Mn)As. Locking between the electron spin with its momentum results in spin galvanic effect. The SGE, following the Rashba Hamiltonian, can be written as ***j***_**c**_ = −*eα*_R_(***z*** × ***S***)/*ℏ*, where ***S*** can be created either by optical and electrical means and is known as non-equilibrium spin density. ***j***_**c**_ is the induced charge current density. This concept was first observed in quantum wells^42,43^ and was used in the context of optical spin manipulation in semiconductors. Quite recently, in a NiFe/Ag/BiAg lateral device, a spin current being pumped from NiFe layer into Ag layer, was converted into a transverse charge current due to SGE which takes place at the Ag/BiAg interface. The ISGE, which manifests itself according to ***S*** = *α*_R_*m*(***z*** × ***j***_**c**_)/*ℏ* is of particular significance when electrically manipulating the magnetization of ferromagnets.

## 4. Spin Hall effects (SHE) and Berry curvature

**Fig. 6:**
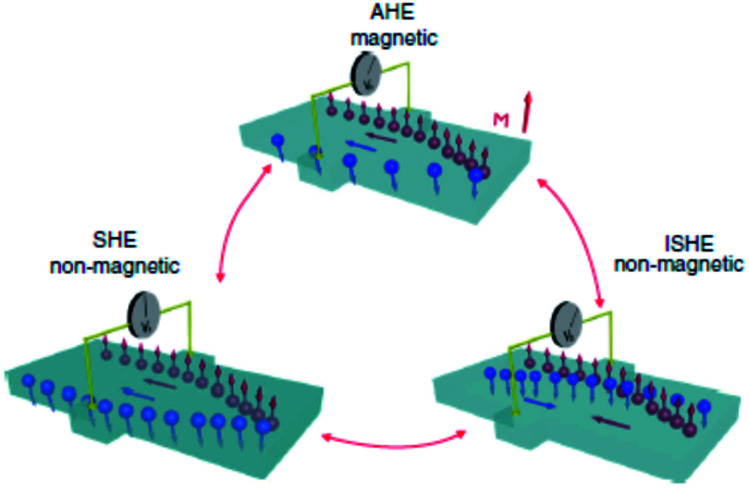
Schematic of illustration of various type of spin-dependent effects. In AHE, an electrical charge current generates a transverse charge current. The SHE is concerned with conversion of an unpolarized charge current to a transverse pure spin current. A pure spin current is converted to a charge current by the phenomenon of ISHE. Figure reproduced with permission from Sinova *et al.*, (2015).^97^

Amongst various possibilities of creating and controlling spin currents, SHE has its own place since its first observation over a decade ago.^21,22,27,44^ In a direct SHE, an electrical current passing through a non-magnetic material with large SOC can generate a transverse pure spin current. In a reciprocal effect, the inverse spin Hall effect (ISHE), a pure spin current through the material generates a transverse charge current. The common factor in both the cases is that the material must possess a reasonably large spin–orbit coupling. The concept of SHE is analogous to another well-known phenomenon, the anomalous Hall effect (AHE), where relativistic spin–orbit coupling produces an asymmetric deflection of charge carriers depending on their spin direction (*cf.*Fig. 6).^45^ The AHE can usually be detected in ferromagnets, where, due to the difference in the population of majority and minority carriers, there is a transverse charge current generated in response to a longitudinal charge current. Incidentally, the generalization of this effect in a non-magnetic material, leading to a generation of pure spin current, was theoretically proposed over four decades ago by Dyakonov and Perel based on the idea of asymmetric Mott scattering.^46^ Hirsch (1999)^47^ and Zhang (2000)^48^ further strengthened this ‘extrinsic’ SHE mechanism from asymmetric Mott scattering whereas Murakami, Nagaosa and Zhang in 2003^49^ as well as Sinova in 2004^50^ proposed the possibility of an ‘intrinsic’ SHE also.

**Fig. 7:**
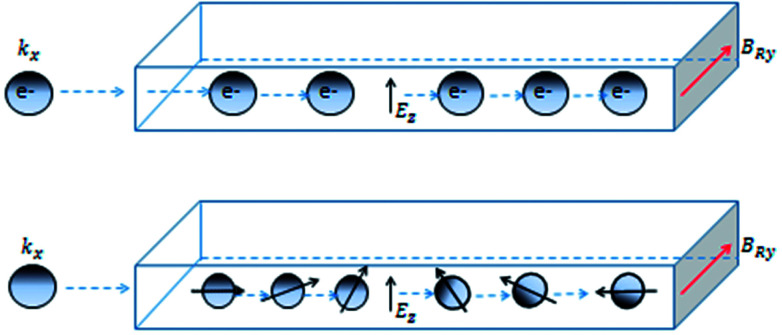
Top: Electrons moving along *x*-direction (*k*_*x*_) in the presence of a perpendicular electric field *E*_*z*_ experience a Rashba field (*B*_R*y*_). Bottom: The spin of moving electrons depicted by arrows, in a Rashba system, precesses around the axis of Rashba field.

To discuss the physics of SHE, let's revert to systems lacking inversion symmetry having *p*-linear Rashba spin–orbit coupling introduced previously eqn (3). This equation describes a Zeeman energy term in which the magnetic field is proportional to the electron momentum *p*. As a result, when motion of electrons is along *x*-axis, they experience momentum-dependent effective field along the *y*-axis, *B*_R*y*_, called the Rashba field as depicted in Fig. 7.

The strength of the Rashba field is given by *B*_R*y*_ = 2*α*_R_*k*_F_/*gμ*_B_. Here *k*_F_ and *g* represent Fermi wavevector and Lande *g*-factor of the carriers in conduction band, respectively. It may happen that when electron spin is not aligned with the Rashba field, spin precession of electrons would occur about the direction of the Rashba field. The frequency of this precession would depend upon the strength of the Rashba field. The bottom panel depicts that, under the influence of the Rashba field along *y*-axis, the spin-polarized electrons injected along *x*-axis, would start precessing along *y*-axis. The frequency of the precession, depending upon the strength of the electric field and hence, the Rashba spin–orbit coupling parameter can be controlled by gate voltages.^7,51–53^ All this happens in the absence of any external magnetic field. An interesting consequence of the emergence of Rashba field is the possibility of polarizing unpolarized electrons (injected along *x*-axis) along the direction of Rashba field (*y*-axis). The presence of Rashba field modifies the velocity of charge carriers according to4
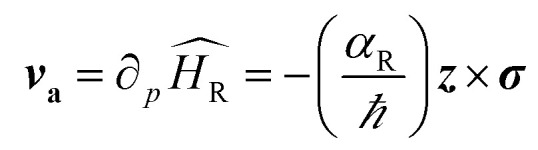


This anomalous velocity bends and distorts the trajectory of the electrons, the direction of distortion depending on the direction of angular momentum (*i.e.*, of spins) resulting in the generation of spin current. This additional velocity, caused by the Rashba field, can be formulated in terms of an effective Lorentz force acting on wavepackets of electrons. The effective magnetic field causing this Lorentz force is called the Berry curvature5
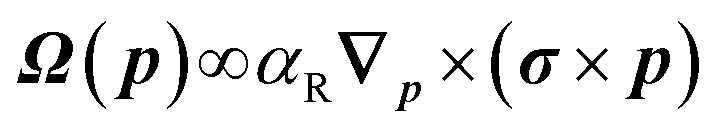
which depends only on geometry of band structure.^54^ The additional velocity induces an off-diagonal conductivity that can be non-zero if time-reversal symmetry is broken. Various properties of Dirac and Rashba materials can be well explained by Berry curvature and its associated Berry phase.

## 5. Coupling of spin Hall effect with magnetization dynamics

There are mainly three different techniques found in literature for studying SHE and its coupling to magnetization dynamics. These three techniques are based on ferromagnetic resonance phenomenon. These are (i) ferromagnetic resonance spin pumping (FMR-SP), (ii) modulation of damping (MOD), and (iii) spin Hall effect-spin transfer torque (SHE-STT). The underlying principle, in all these three techniques, is similar. With reference to a bilayer FM–NM structure, the FM layer is used to inject or absorb the spin current into or from the NM layer and investigate the effect of magnetization dynamics.

### 5.1. Ferromagnetic resonance spin pumping

In FMR-SP, a spin current in injected from the ferromagnetic layer into the non-magnetic (NM) one. The injected spin current does not accompany charge current. But this spin current can easily be detected by electrical means, thanks to the phenomenon of inverse spin Hall effect (ISHE) in the non-magnetic material. In ISHE, a spin current injected in a NM layer gets converted to a charge current. The efficiency of conversion of a charge current to spin current is quantified by spin Hall angle. In the process of spin injection from a FM layer into the NM, angular momentum is lost in the FM, hence the FMR-SP leads to broadening of FMR linewidth.^55–57^ Incidentally, the backflow of spin current into the ferromagnetic layer generates a dc voltage and this can also be used to detect spin pumping.^58^ This dc voltage due to backflow of spin current has been observed by several groups.^59,60^ In the theory of ferromagnetic resonance spin pumping, Tserkovnyak, Brataas and Bauer (2002)^61^ and Tserkovnyak, Brataas and Halperin (2005)^62^ showed that a precessing magnetization in a ferromagnet (due to external field) generates a spin current density (time dependent) at the FM–NM interface.

The spin current, generated at the interface, can propagate into the non-magnetic material and decays in a length scale given by the spin-diffusion length *λ*_sd_ of the NM. The time dependent spin current is given by6
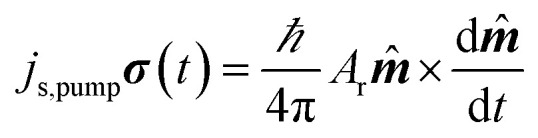
here ***m̂*** is the unit vector of magnetization and ***σ*** represents the polarization of the spin current. *A*_r_ is the spin-pumping conductance of the particular sample. In systems with strong spin orbit coupling, the spin-diffusion length can be as short as few atomic layers. Proximity effects as well as interface roughness can contribute to non-transparent FM–NM interface.

*A*_r_ is related to the complex spin-mixing conductance, a quantity that arises from scattering matrix theory based on spin-conserving channels and no spin losses at the interface. Theoretical *ab initio* calculations show that only the real part of spin mixing conductance dominates the physics at the FM/NM interface. Under this approximation, Tserkovnyak, Brataas and Bauer^63^ showed7
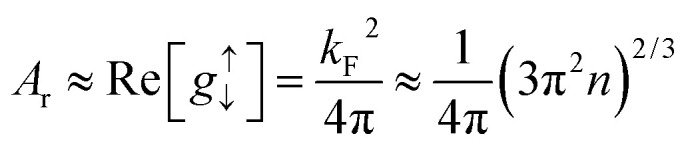
where *k*_F_ and *n* are the Fermi wave vector and electron density in the NM respectively.

As the magnetization precesses inside the FM layer, the spin current injected from FM into NM is time dependent. But this ac spin current, when averaged over time, has an associated dc component given by8
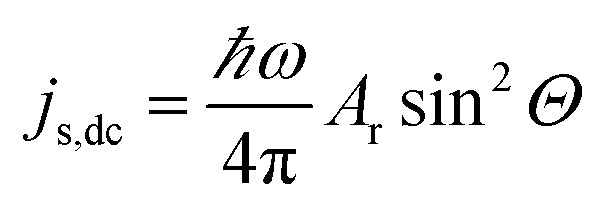
where *ω* represents the driving frequency and *Θ* represents the cone angle of precession. (In fact, the projection of the spin current along the static magnetization direction of the ferromagnet gives the dc component of spin current *j*_s,dc_.) Under the assumption of the NM being an ideal spin-sink, the spin pumping conductance *A*_r_ would be equal to the spin mixing conductance *g*^↑^_↓_. However, in systems where the thickness of NM is of the order of spin diffusion length *λ*_sd_, there would be spin accumulation in the NM during spin pumping from FM. This spin accumulation will, in turn, create a spin backflow *j*_s,back_***σ***(*t*).^58,59,61^

The spin accumulation with the non-magnetic material is governed by the equation9
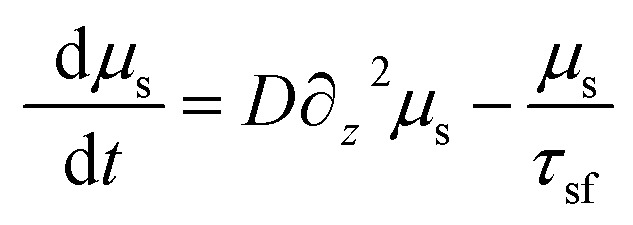
where *μ*_s_ = *μ*^↑^ − *μ*^↓^ is the spin-dependent chemical potential, *D* is the diffusion co-efficient and *τ*_sf_ is the spin-flip scattering time.

In the NM layer, the spin current decays away from the interface due to contributions from spin diffusion process and also spin flip scattering. If *z*-axis denotes the direction normal to the interface (interface located at *z* = 0), the *z*-dependent spin current in the NM is given as10
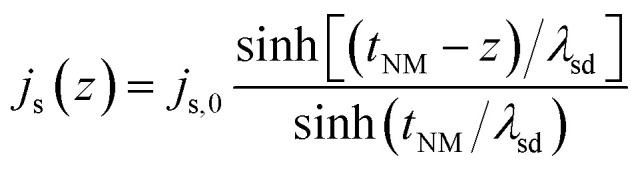
where *t*_NM_ denotes thickness of the non-magnetic layer.^64,65^

If we include the backflow current density ***j***_**s**,back_, then the following equation needs to be solved for the total spin current crossing the interface (at *z* = 0).11
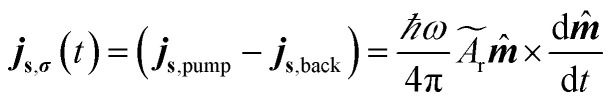
where 
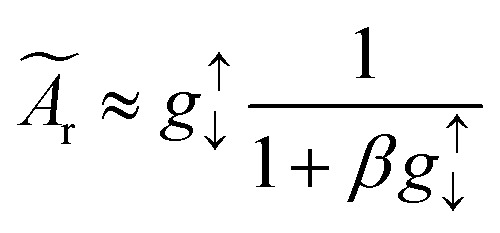
 is the effective spin mixing conductance which is reduced by backflow factor *β* given by12
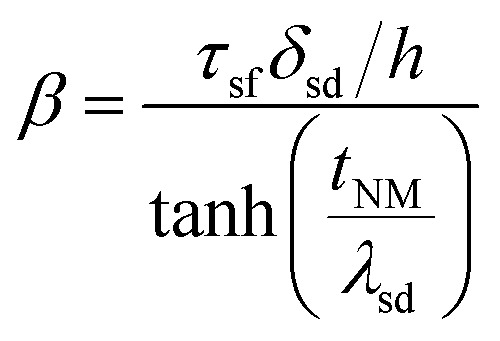
where *δ*_sd_ is the effective spin-flip scattering energy.

**Fig. 8:**
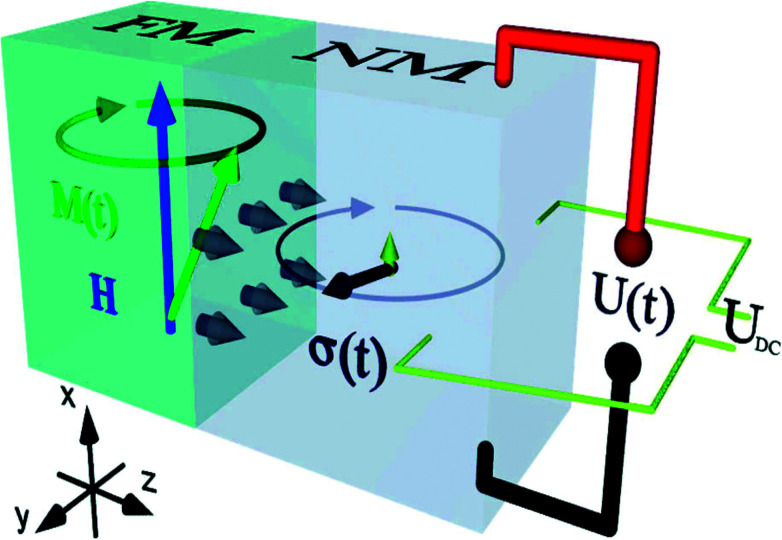
Generation of spin current (grey arrows) at the interface of a FM–NM layer by spin pumping (SP). Generation of spin current (represented by grey arrows) by spin pumping at the interface of a FM–NM bilayer. The dark grey arrow indicating the spin polarization of this spin current rotates in the *y*–*z* plane. The small upward arrow represents dc component of time-dependent spin current. Both these components lead to charge currents in NM layer. Figure reproduced with permission from Wei *et al.*, (2014).^98^

The detection of the net spin current across the non-magnetic layer can be made electrically by inverse spin Hall effect (ISHE) as had been demonstrated by Saitoh *et al.* in 2016.^66^ By measuring the Hall voltage induced by the spin current, the spin Hall angle (*α*_SH_) of the material can be determined by13
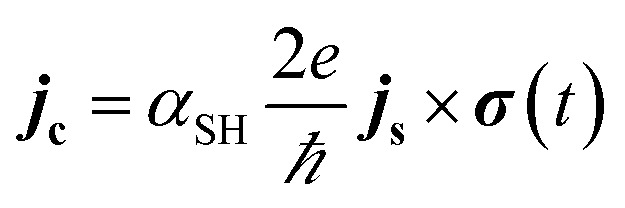
here the spin current density vector ***j***_**s**_ points perpendicular to the interface of NM-FM. The spin-polarization direction ***σ***(*t*) is a time-varying quantity. In Fig. 8, the propagation direction of spin current is along *z*-axis and its polarization is along *x*-axis. Hence one has to consider the dc voltage along the *y*-direction to compute the charge current due to spin pumping. This is given by14
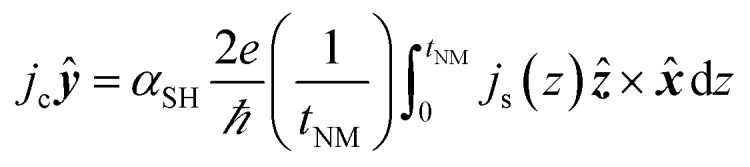


This magnitude, according to Azevedo *et al.*,^65^ had been calculated to be15
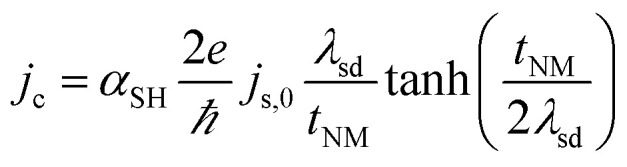
which actually contributes to ISHE voltage as a result of spin current resulting from spin pumping.

**Fig. 9:**
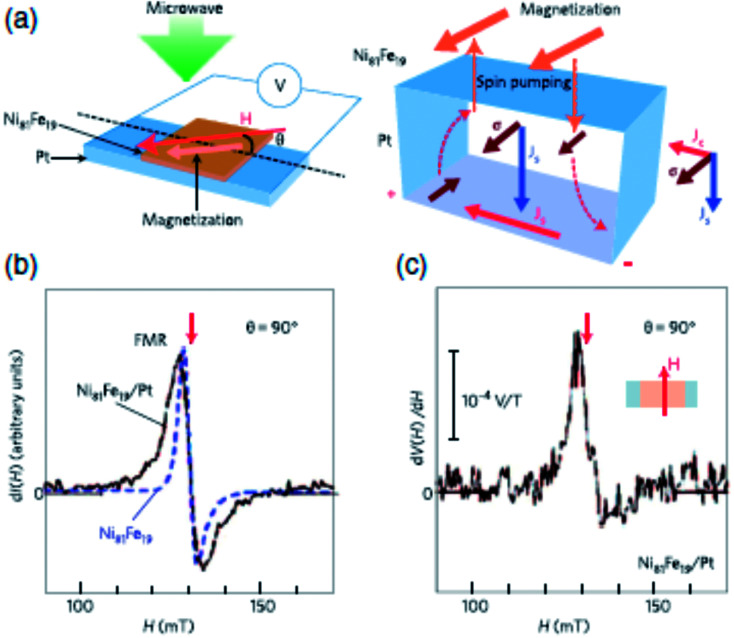
Inverse spin Hall effect (ISHE) in a metal device with spin injection from a FM layer by spin pumping through FMR (FMR-SP). (a) Schematic of NiFe/Pt system used in the study for investigating ISHE. (b) FMR studies showing magnetic-field dependence of the FMR signal for the NiFe/Pt bilayer film and a bare NiFe film. *I* denotes the microwave absorption intensity. (c) FMR signal in the form of magnetic field dependence of potential difference between electrodes on Pt layer, d*V*(*H*)/d*H*. Figure reproduced with permission from Saitoh *et al.*, (2006).^66^

The above equation is used to convert the spin current into measured voltage. It is common that while performing FMR-spin pumping experiments, voltages arising due to anisotropic magnetoresistance (AMR) and inverse spin Hall effect (ISHE) also appear. Thus, great care needs to be taken to eliminate these two contributions. In the original experiments by Saitoh *et al.* (2006),^66^ as shown in Fig. 9, the ferromagnetic resonance spectrum of NiFe/Pt bilayer sample is compared with that of the reference NiFe sample.

The linewidth of FMR spectrum of NiFe/Pt sample is larger than NiFe sample which demonstrates the spin pumping into the Pt layer through the interface. The dc voltage, induced by spin pumping, along the plane of interface was also measured simultaneously. Saitoh *et al.* (2006)^66^ and Ando *et al.* (2008)^67^ found that the signal is only present when the spin polarization vector is perpendicular to the electric field measured across the sample.

### 5.2. Spin Hall effect modulation of magnetization damping

In modulation of damping experiments, the spin current induced due to SHE in a non-magnetic layer (by directly passing dc electric current) is used to modify the damping in the ferromagnetic layer that has already been in the state of ferromagnetic resonance. The dc spin current injected across the FM–NM interface leads to a damping or anti-damping-like torque acting on the precessing magnetization of the ferromagnetic layer. Modulation of damping is observed from the FMR linewidth analysis as functions of external magnetic field and dc charge current.

**Fig. 10:**
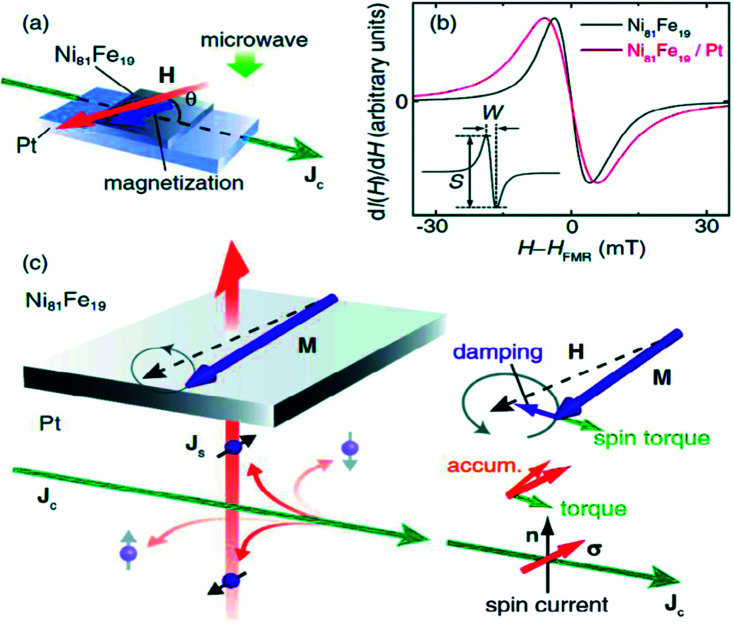
(a) Illustration of a modulation of damping (MOD) experiment in a NiFe film to determine spin Hall angle. The external magnetic field and current density are represented by *H* and *J*_c_ respectively. (b) Magnetic-field dependence of the FMR signal for a NiFe/Pt bilayer film and a pure NiFe film. The line broadening in NiFe/Pt sample is due to spin pumping (SP). (c) Spin Hall and spin torque effects in NiFe/Pt thin films with *M*, *J*_s_, and ***σ*** denoting the magnetization, the flow direction of the spin-current density, and the spin-polarization vector of the spin current, respectively. Figure reproduced with permission from Ando *et al.*, 2008.^67^

The Fig. 10 below shows a typical modulation of damping experiment proposed by Ando *et al.* (2008).^67^ Here the FM–NM layer comprising of a Py/Pt sample is placed in a microwave cavity (resonance frequency 9.4 GHz) and is subjected to a rf driving magnetic field. By adjusting the external dc magnetic field, the bilayer can be brought to a FMR state. The direction of external magnetic field was at an angle *θ* w.r.t to the direction of current flow. The effect of spin pumping in the Py/Pt bilayer can be observed from FMR linewidth broadening as compared to that of Py layer only. The spin current, generated due to SHE, enters the magnetic NiFe film. The propagation direction of the spin current is perpendicular to the interface whereas the direction of spin polarization of spin current depends upon the direction of charge current flow. The spin current exerts a torque on the precessing magnetization and this torque can either add to or oppose the damping torque already acting on the magnetization of FM layer. Further it was found that the effect is maximized when the external magnetic field points perpendicular to the direction of current flow.

The effect of injected spin current on the precessing magnetization can be modeled by the additional contribution from spin transfer torque (STT) in addition to contribution of spin pumping in Landau–Lifsitz–Gilbert equation.^67,68^ This is given by17
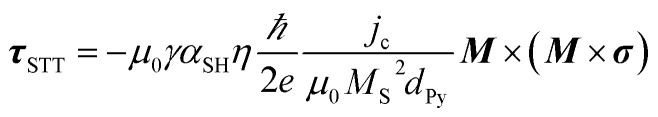
here *d*_Py_ is the thickness of the magnetic Py layer and ***τ***_STT_ is the torque associated with STT. Ando et *al.*,^67^ also observed that STT generated by the spin current traversing the FM–NM interface due to SHE alters the FMR linewidth when the current flow direction and the external magnetic field enclose an angle of 90° whereas there is no effect for collinear orientation consistent with theoretical prediction.

Similar experiments related to modulation of magnetization damping by SHE have been performed by Demidov, Urazhdin, Edwards, Demokritov (2011)^69^ and Demidov, Urazhdin, Edwards, Stiles et *al.*, (2011)^70^ using Brillouin light scattering technique. The important findings were the control of FMR linewidth in ferromagnetic layer by employing SHE. In suitable nanostructured (nano pillars, nano-contact *etc.*) materials, the application of large enough charge current density lead to coherent oscillations in the ferromagnetic nanostructure and such spin Hall nano-oscillators are of particular interest in research of tunable microwave sources.^71–75^

### 5.3. Spin Hall effect-spin transfer torque (SHE-STT)

In the process of SHE-STT, a spin current, generated due to SHE in non-magnetic material, is used to transfer spin angular momentum to the ferromagnetic layer. This process leads to a torque on the magnetic moments of FM layer. These experiments consist of sending ac currents along the interface of a bilayer FM–NM sample which can create an rf excitation of the magnetization in FM. Now if the FM is in the state of ferromagnetic resonance, this additional torque due to the transfer of spin current into the FM layer can be detectable by FMR linewidth measurements. For conventional systems involving spin transfer torque, an electrical current is sent perpendicular to the stack containing two ferromagnetic electrodes. The transfer of angular momentum, from one FM layer to another, has been observed by Ralph and Stiles (2008).^76^ SHE-STT experiments, on the other hand, rely on perpendicular spin current (perpendicular to the plane of interface) due to in-plane charge current in the attached non-magnetic material by the phenomenon of SHE. In both the modulation of damping as well as in SHE-STT experiments, the torques generated in the ferromagnetic layer by SHE in the non-magnetic material would be on top of ISGE related spin–orbit torques that are present in inversion asymmetric FM–NM interface.^38,41,77^ As such, the spin Hall angle should be a parameter due to all the torques generated. Therefore it must be considered as effective spin Hall angle for the specific bilayer system.

**Fig. 11:**
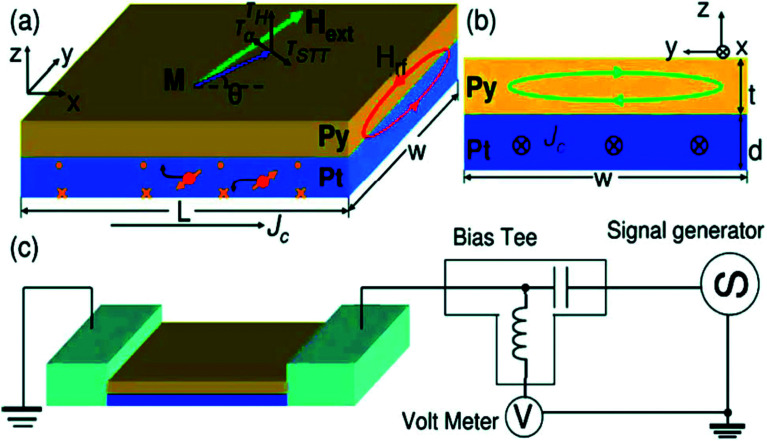
Schematic illustrating spin transfer torque induced by spin Hall effects due to the rf current through NM (Pt).(a) The system is Pt/Py bilayer film. Along with STT, the torque due to Oersted field as well as damping torque is also shown. (b) The dimensions of the sample and the Oersted field due to a current flowing through FM. (c) Schematic of electrical measurement of the same Pt/Py system. Figure reproduced with permission from Liu *et al.*, (2011).^68^

As reproduced in Fig. 11, Liu et *al.*, (2011)^68^ applied a high frequency (microwave range) charge current in the plane of a NiFe/Pt bilayer and observed ferromagnetic resonance in NiFe. Because of the SHE in the non-magnetic material (Pt), a transverse spin current is injected into the ferromagnetic layer, and consequently, an oscillatory spin transfer torque (STT) acts on the magnetic moments of FM layer. The oscillatory magnetization in the ferromagnet leads to an oscillatory anisotropic magnetoresistance again leading to an oscillatory resistance. This high frequency resistance mixes with the rf current and leads to a detectable dc voltage across the device. This voltage is usually picked up by a bias tee.

In these experiments, the external magnetic field, after fixing it at an angle of 45°, is swept in the plane of the film to achieve FMR condition. The different torques acting on the magnetization of the ferromagnetic layer is depicted in the figure. Typically, the torques include the spin transfer torque (STT) due to spin Hall effect (SHE) in the NM, torque induced by the Oersted field due to the rf current and the usual damping torque due to spin pumping.

Typically, the dc voltage response for the bilayer device is modeled by Landau–Lifstiz–Gilbert equation which takes into account all possible types of torques. The mixing voltage contains contributions of symmetric and antisymmetric Lorentzian lines. Liu *et al.*, in 2011^68^ showed that the resonance properties of the mixing voltage is a quantitative measure of the spin current absorbed by the ferromagnetic layer, and of the spin Hall angle. In the same work it was also shown that the ratio of the symmetric to antisymmetric components of the resonance curve is related to the spin Hall angle.^68^ Using various combinations of FM and NM, the same method has been used to determine the spin Hall angle.^68,72,78^

## 6. Future directions, challenges and applications

The field of SHE, by bringing non-magnetic transition metals into the picture, has given a new dimension to it. It is well known how transition metals have played their role in spintronics – both in basic research as well as in application point of view. When subjected to an applied electric field (hence bringing it to out-of-equilibrium) in some non-magnetic transition metals, sufficient flux of spin angular momentum can be created by virtue of SHE or inverse spin galvanic effect (ISGE). This spin flux is strong enough to reorient, and in some cases, reverse the magnetization of the adjacent transition metal ferromagnet. New concepts of writing information in magnetic tunnel junctions and domain wall based spintronic devices, are under intense development. Industries are looking for high performance and high density memories, notably the STT-MRAM and racetrack memories. The integration of semiconductor logic circuits with STT magnetic memory is also another focus to enhance functionality and reduce energy. Magnetic tunnel junctions (MTJ) are the central elements for the fast read out of memory and logic devices.

### 6.1. Magnetic random access memories (MRAM)

In hard disk drives and magnetic memories, magnetization switching is the most fundamental process. Magnetic tunnel junctions are used as storage devices in magnetic random access memories (MRAM), where the magnetic fields are used to write information as functions of magnetization direction in one of the two FM electrodes of MTJ. Keeping in mind that miniaturization of magnetic bits results in being more susceptible to thermal fluctuations, the coercive field must be increased. This means that currents that are required to generate magnetic fields for magnetization switching, must also be large after miniaturization. In contrast, in spin-transfer torque devices, switching currents scales up in direct proportion to the energy barrier for magnetization reversal. The switching current does not scale with the dimension of magnetic bit. The importance is that the technologies based on spin transfer (and not magnetic fields from charge currents) are more welcoming and dimension of magnetic bits continue to shrink. In 2005, a 4-kbit of STT-MRAM was first reported with CoFe(B)–MgO MTJ.^79^ Demonstrations of 2 Mbit,^80^ 32 Mbit^81^ and 64 Mbit^82^ STT-MRAM followed after this. All these works employed bcc CoFe(B)–MgO^82–86^ magnetic tunnel junctions to achieve high MR ratios together with STT switching.^87–89^ The scalability of magnetic tunnel junctions is the key issue for making high density MRAM devices. MRAM devices based on STT require a tunneling magnetoresistance ration (TMR) greater than 100%, good thermal stability of magnetic storage element and low switching current. The thermal stability is given by *Δ* = *E*/*k*_B_*T*, where *E* is the energy barrier between parallel and antiparallel states of magnetization. Ideally, the thermal stability needs to be 60% or greater for long term data retention. Since *E* is the product of anisotropy energy density *K* of the magnetic storage element and the volume *V*, this means that higher *K* is required to maintain the required stability factor as the dimension of the magnetic bit is shrunk. This justifies the emergence of thin films with strong perpendicular magnetic anisotropy for use as electrodes in MTJ. The threshold current on the other hand, which needs to be reduced with the size of the device, is proportional to the product of *E* and Gilbert damping constant *α*. This makes it necessary to design a material having high anisotropy constant *K* and low *α*. Typically, a CoFeB–MgO based perpendicular MTJ exhibits a high TMR ratio (>100%), switching current as low as 50 μA and *Δ* = 40 at dimension of 40 nm in diameter.^90^ In MRAM applications, switching speed is one of the key issues. In collinear MTJ devices, the initial current induced spin torque is zero (spin torque proportional to ***m*** × (***m*** × ***I***_**s**_), ***I***_**s**_ being the spin current). So thermal fluctuations and misalignments of layer magnetization are required to originate switching, leading to undesirable delays and broad switching distributions. This is the reason also for the exploration of MTJ stacks with non-collinear magnetization wherein a perpendicularly magnetized polarizing layer is combined with an in-plane magnetized MTJ.

**Fig. 12:**
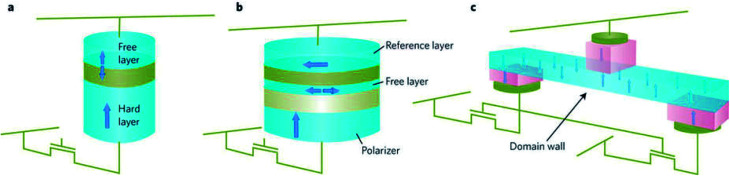
Three different concepts for magnetic random access memories. (a) Two-terminal collinear device. (b) A device with three magnetic layers that includes a perpendicular magnetized spin-polarizing layer, an in-plane magnetized free layer and reference layer. (c) Three-terminal device. The devices are integrated with CMOS select transistors as illustrated. In (a) and (b), short current pulses that flow perpendicular to the layer planes are used to change the magnetic state of a free layer and write information. In c, an in-plane current is used to move a domain wall in a nanowire. In each case a magnetic tunnel junction is used to read the magnetic state of the storage magnetic element. Figure reproduced with permission from Brataas *et al.*, (2012).^99^

Three-terminal STT-MRAM bit cells, with separate read and write contacts, are also looking promising nowadays. Usually, high current densities are required for writing which can lead to a breakdown of MTJ in two terminal devices. Also, reading the magnetic state requires a current flow through the MTJ which can also change the magnetic state. By using separate read and write contacts – a low impedance contact for writing and an MTJ for read-out, the above issue can be addressed. In Fig. 12(c), current induced domain wall motion is used to write data and the domain wall position, that is, to the left or right of MTJ is read out with a MTJ.

### 6.2. Integrated circuits with logic-in-memory

A new way of designing integrated circuits, employing a logic-in-memory architecture, involves MTJ based non-volatile memories distributed over the logic plane. Since MTJs involve use of metals with insulating tunnel barrier and not any semiconductors, they can be integrated in the interconnection layer above the semiconductor transistor layer. With this new architecture, the two main obstacles hindering the performance increase of IC – power consumption (static and dynamic) and interconnection delays. With this integration, the MTJ based devices have the attributes of non-volatility, high speed, high endurance, scalability and low voltage operation. The following are the advantages: reduction of standby power, interconnection delay between memory and logic (von Neumann bottleneck) greatly reduced and reduction in number of transistors involved. Experimental circuit analogs of MTJ being integrated with CMOS based circuits have been demonstrated.^91–93^

### 6.3. Racetrack memory

**Fig. 13:**
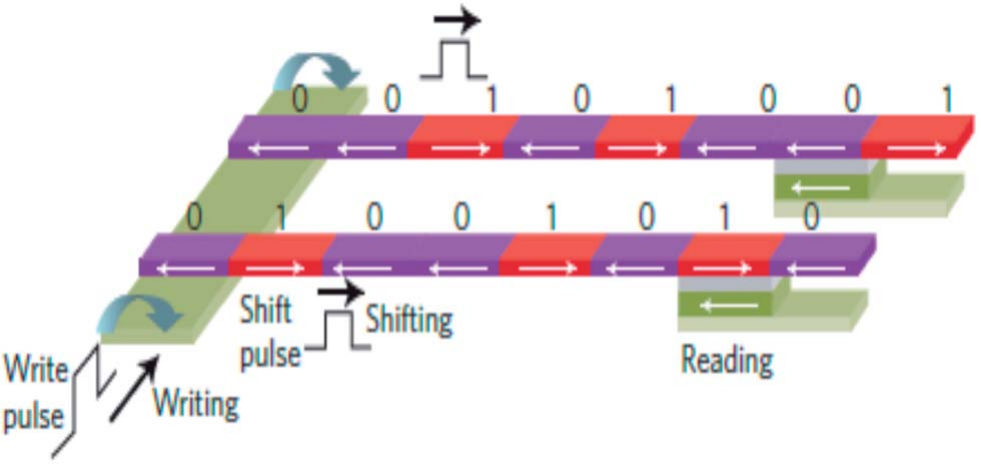
Schematic of racetrack memory. A write pulse on the left produces magnetic fields to reverse the magnetization in the desired direction. A shift pulse exerts spin-transfer torque and shifts all the domain walls in the same direction. Reading is done with a magnetic tunnel junction. Figure reproduced with permission from Thomas *et al.*, (2011).^95^

Recently, current induced domain wall motion based high-density memory has been proposed and developed (*cf.*Fig. 13). The technology has been coined as ‘racetrack memory’. Here the domain walls are generated by local magnetic fields in nanowire systems. They are moved by current pulses and read by MTJ based sensors.^94^ The principle involves shifting of the domain walls in the same direction by short current pulses in the nanowire systems and reading with MTJ based sensors. Since ten to hundreds of domain walls can be stored in single nanowire, a 3D integration of racetrack memory offering a denser, faster, more robust and less expensive mass storage, is expected. A six bit shift register using Co/Ni nanowires with perpendicular anisotropy has been reported.^95^ A racetrack memory array with CMOS circuitry and using MTJ readout has also been demonstrated using permalloy nanowires.^96^

Amongst non-magnetic materials, Ta, W, Ir, and Pt have large SHE due to large spin–orbit coupling in these heavy elements. But the proper description and microscopic understanding of SHE structures in the strong spin–orbit coupling regime (in above heavy transition metals) is amongst key challenges in the field of SHE. A large number of recent studies of SHE in transition metals may give us an impression that the field is forgetting its semiconductor roots. It is well known that some of the robust ferromagnets are dense-moment systems and the magnetization switching in such systems require moderately large current densities if SHE generated spin current is envisioned for magnetization manipulation. As the value of spin Hall angle in transition metals is larger than that in semiconductors, hence, the transition metal with large conductivity may better fit for application in comparison to semiconductors. Semiconducting materials can still be foreseen playing a vital role in spintronics with ferromagnets. Spin Hall effect based spin current in a transition metal, is only used as an efficient spin current generator and is rarely used for manipulating spin in a non-magnetic spin Hall effect system. The spin-diffusion length in non-magnetic transition metals with a high spin–orbit coupling is exceedingly small, of the order of few nanometers, making the possibility of spin manipulation along the non-magnetic transport channel a minimum. For semiconductors, electrical manipulation of output SHE signal can be made – for example, a gate electrode can be used to control coherent spin precession along the channel and non-linear intervalley transport can strongly enhance spin Hall angles close to the values of heavy metal counterparts. Large SHE requires large spin–orbit coupling which tends to suppress spin coherence or spin diffusion length. Semiconductors, on the other hand with simpler electronic structure and modest spin–orbit fields offer ways to circumvent this problem. For example, a proper tuning of Rashba and Dresselhaus fields can enhance the spin coherence in the presence of spin–orbit coupling. Another emerging field involving the combination of optical selection rules with SHE is opto-spintronics which mainly include spin voltaic cells, optical spin-torque structures, *etc.* It is worth mentioning here that this field of research is evolving and is expected to grow rapidly in the coming few years.

## Conflicts of interest

There are no conflicts to declare.

## Supplementary Material
